# *Staphylococcus epidermidis* Pathogenesis: Interplay of *icaADBC* Operon and MSCRAMMs in Biofilm Formation of Isolates from Pediatric Bacteremia in Peshawar, Pakistan

**DOI:** 10.3390/medicina58111510

**Published:** 2022-10-24

**Authors:** Saghir Ahmad, Hazir Rahman, Muhammad Qasim, Javed Nawab, Khalid J. Alzahrani, Khalaf F. Alsharif, Fuad M. Alzahrani

**Affiliations:** 1 Department of Microbiology, Abdul Wali Khan University, Mardan 23200, Pakistan; 2 Department of Microbiology, Kohat University of Science and Technology, Kohat 26000, Pakistan; 3 Department of Environmental Sciences, Kohat University of Science and Technology, Kohat 26000, Pakistan; 4 Department of Clinical Laboratories Sciences, College of Applied Medical Sciences, Taif University, Taif 21944, Saudi Arabia

**Keywords:** *Staphylococcus epidermidis*, pediatric, biofilm, *icaADBC* operon, MSCRAMMs

## Abstract

*Background and Objective*: *Staphylococcus epidermidis* is an opportunistic pathogen from pediatric bacteremia that is commonly isolated. Biofilm is the major virulence factor of *S. epidermidis*; however, the role of biofilm determinants in biofilm formation is highly contradictory and diverse. The current study aimed to investigate the role of polysaccharide-dependent and polysaccharide-independent pathogenic determinants in biofilm formation under physiological stress conditions. *Materials and Methods*: The isolates (*n* = 75) were identified and screened for the *icaADBC* operon, *IS256,* and an array of MSCRAMMs (Microbial Surface Component Recognizing Adhesive Matrix Molecules) through PCR analysis. The activity of the *icaADBC* operon was detected by Congo red assay, and the biofilm formation was analyzed through microtiter plate assay. *Results*: *S. epidermidis* isolates produced biofilm (*n* = 65; 86.6%) frequently. The *icaA* was the major representative module of the actively expressing *icaADBC* operon (*n* = 21; 80.7% sensitivity). The MSCRAMMs, including *fbe* (*n* = 59; 90.7%; *p* = 0.007), and *embp* (*n* = 57; 87.6%; *p* = 0.026), were highly prevalent and associated with biofilm positive *S. epidermidis*. The prevalence of *icaADBC* operon in biofilm positive and negative *S. epidermidis* was not significant (*n* = 41; 63%; *p* = 0.429). No significant association was found between *IS256* and actively complete *icaADBC* operon (*n* = 10; 47.6%; *p* = 0.294). In the presence of 5% human plasma and glucose stress, *S. epidermidis* produced a strong biofilm (*n* = 55; 84.6%). *Conclusion*: The polysaccharide-dependent biofilm formation is significantly replaced (*n* = 21; 28%; *p* = 0.149) by a polysaccharide-independent mechanism (*n* = 59; 90.7%; *p* = 0.007), in which the MSCRAMMs might actively play their role. The fibrinogen-binding protein and extracellular matrix-binding protein might be potential anti-biofilm drug targets, markers of rapid diagnosis, and potential vaccine candidates of *S. epidermidis* involved in pediatric bacteremia.

## 1. Introduction

*Staphylococcus epidermidis* is an opportunistic pathogen in the Coagulase-Negative Staphylococci (CONS) group of pathogens most frequently recovered from clinical care settings. Its incidence has a parallel association with advancements in medicines, especially with foreign body-related devices [1]. Biofilm, a three-dimensional complex exopolysaccharide (EPS), is a predominant virulence factor in *S. epidermidis* [2].

Polysaccharide Intercellular Adhesin (PIA) is the significant EPS type in *S. epidermidis* that plays a vital role in biofilm formation by adhering cells to inanimate surfaces and is encoded by the *icaADBC* operon locus [3]. This operon comprises four key components. The first two genes, including *icaA* and *icaD,* are the leading players in the synthesis of PIA, also called Poly-*N*-acetylglucosamine (PNAG), the chief product of the first module, *icaA*. Enzymatic activity of *icaA* significantly increases in the presence and co-expression of *icaD* [4]. Nosocomial behavior, e.g., extensive persistence in hospital environments, is associated with strains carrying *icaA* and *mecA* [3]. 

*IS256* regulates the *icaADBC* operon by reversible insertion into the *icaA* module of the *icaADBC* operon, leads to the appearance of a phase-variation phenotype of decreased or abolished synthesis of PIA [5,6]. *S. epidermidis* can switch to a protein-dependent biofilm formation upon insertion and subsequent disruption of the *icaADBC* operon [7]. It indicates that the presence of *icaADBC* operon cannot ensure the active synthesis of PIA, as it is under tight regulation. *IS256* is also considered as a marker of pathogenicity and is associated with *S. epidermidis* of infection origin [8]. 

However, initially assumed as crucial for biofilm formation and the principal component of EPS [9,10,11], the concept of a PIA-independent mechanism led to the discovery of a giant extracellular matrix-binding protein (Embp). Embp is a cell wall-associated (CWA) surface protein and a member of the Microbial Surface Component Recognizing Adhesive Matrix Molecules (MSCRAMMs), which binds with the plasma fibrins on implant surfaces [12]. Hence, polysaccharide dependence was considered non-essential for virulence [13]. SdrG/fbe is another highly studied MSCRAMM, which binds specifically to fibrinogen [14] and is involved in strong and more elastic biofilm formation in *S. epidermidis* [15]. Like the fate of PIA-dependent biofilm formation, the PIA-independent biofilm mechanism and role of MSCRAMMs in biofilm formation have also been investigated recently, especially in biofilm conditions mimicking the in-vivo environment [16].

Despite the frequent isolation in pediatrics, no data is available about the virulence determinants of *S. epidermidis* in the pediatrics Departments of Tertiary Care Hospitals in Pakistan. Previous researchers have studied the association of polysaccharide-dependent and polysaccharide-independent virulence determinants with biofilm formation, however, hazy association and variations, e.g., interpretation of slime as biofilm, emphasis on polysaccharide-dependent biofilm formation, and geographical variations in MSCRAMMs associated with invasive strains, are frequently observed. Therefore, there is an urgent need to trace the pathogenic behavior of *S. epidermidis* in this geographical locality. This study investigated the PIA-dependent (*icaADBC* operon-dependent) and PIA-independent (MSCRAMMs-dependent) biofilm-forming potential of *S. epidermidis* isolated from pediatric bacteremia. 

## 2. Materials and Methods

### 2.1. Sampling and Identification

One thousand blood culture samples were obtained from patients in the Pediatric Department and were sent to the Microbiology section from March 2021 to August 2021. About 150 CONS were isolated in pure culture following the recommended guidelines of blood culture isolation [17]. *S. epidermidis* was biochemically identified through catalase, coagulase and DNAse tests, performed on 24 h grown pure culture isolates. For catalase test, a pure colony was mixed with a drop of 3% hydrogen peroxide on a clean, dry glass slide. Rapid evolution of oxygen (bubbles formations) was considered as catalase positive. The slide coagulase test was performed to detect the presence/absence of Staphylococcal surface clumping factors (bound coagulase) by emulsifying a pure colony in normal saline on a clean, dry glass slide until the appearance of milky suspension. A drop of undiluted human plasma was placed and stirred into the suspension. Absence of visible clumps within 10 s was considered as negative. Slide coagulase negative isolates were further confirmed by tube coagulase test. Several colonies were emulsified in 1 mL of diluted plasma (1 mL plasma + 5 mL, 0.85% normal saline) in a small sterile test tube and incubated at 35 °C in water bath for 4 h. The tube was observed after 1, 2 and 4 h. Absence of clot formation was considered as tube coagulase negative. For DNAse test, the isolates were incubated for 24 h at 37 °C on DNAse agar, followed by pouring of 15 mL, 1 N HCL. Excess acid was removed and cleared zones around growth was considered positive, while no zone formation was considered as negative [18]. Molecularly identified *Staphylococcus aureus* and *S. epidermidis* were used as controls in each biochemical identification step. The catalase positive, while coagulase and DNAse negative isolates were further molecularly identified through amplification of the *rdr* gene’s species-specific target region (Table 1). The Medical Institute-Research Ethics Committee approved the study (RMI/RMI-REC/Approval/8, 8 December 2020).

### 2.2. Phenotypic Analysis

#### 2.2.1. Congo Red Assay for *icaADBC* Operon Activity

All isolates were cultured on Congo red agar to check the activity of *icaADBC* operon, including the synthesis of PIA and its subsequent actual contribution to biofilm formation. The Congo red assay was performed as previously described [19] with minor modifications in the formulation. All isolates were cultured on nutrient agar supplemented with 0.08% Congo red dye and 5% sucrose as a stress inducer and precursor of PIA. The isolates were incubated at 37 °C for 24 h under aerobic conditions. The results were interpreted as previously described [20] with minor modifications. Only pure black and dry cultures/colonies were deemed positive, and the operon was considered actively expressing its product, the PIA/PNAG. Isolates producing either red color colonies or b/w color morphologies, e.g., red, deep red, intermediate, and Bordeaux, were all considered negative to rule out false-positive results caused by defective and missing operon.

#### 2.2.2. Biofilm-Formation Assay under Plasma Stress

A standard protocol of biofilm formation [21] was followed with modifications. Nutrient broth was humanized with 5% filtered sterilized human plasma and 5% glucose to induce the MSCRAMMs machinery and *icaADBC* operon [22], mimicking in-vivo conditions, *S. epidermidis* encounters during infections in high-share environments, e.g., 0.2 µL of overnight-growing bacteremia cultures were transferred to the microtiter plate well containing 198 µL of humanized nutrient broth. The transfer was carried out to obtain an approximate inoculum of 5 × 10^6^ cells/well incubated at 37 °C for 48 h. Before processing for biofilm measurements, OD of the 48 h grown cultures in 96-well microtiter plate was measured at 600 nm to check the growth. Culture OD ≤ 2ODC was considered as a slow grower (SG). The biofilms were heat-fixed at 60 °C after washing, stained with 0.2% crystal violet for 5 min, and eluted with 33% acetic acid, followed by OD measurements at 570 nm (BioTek Instruments Inc., EL×800, Winooski, VT, USA). The microtiter plate assay was repeated thrice with triplicates well/each isolate (31 isolates/batch) along with a negative control. OD was calculated by subtracting the mean of the OD triplicate from the ODC (Control).

### 2.3. Detection of icaADBC Operon, MSCRAMMs, and IS256

DNA from *S. epidermidis* was extracted per the manufacturer’s instructions (Chelax^®^ 100, Biorad, Contra Costa County, CA, USA). All the strains of *S. epidermidis* were screened for *icaADBC* operon by detecting *icaA* and *icaD*. For polysaccharide-independent virulent determinants, the isolates were screened for an array of MSCRAMM genes, including serine-aspartate dipeptide repeats G/fibrinogen binding protein (*SdrG/fbe*), extracellular matrix binding protein (*embp*), enolase/laminin-binding protein (*eno*), accumulation associated protein (*aap*), biofilm homologous protein (*bhp*), and biofilm-associated protein (*bap*). The isolates were also screened for *IS256* to check its association with *icaADBC* operon activity (PIA synthesis) in Congo red assay. All the molecular screening was performed through a series of multiplexed PCR (SC300G-R2, Kyratec, Mansfield, Australia) using a master mix (DreamTaq Green PCR Master mix 2(×), Thermo scientific, Vilnius, Lithuania). The amplicons and a 100 bp DNA marker (GeneRuler, Thermo scientific, Lithuania) were analyzed in gel electrophoresis followed by visualization in a UV transilluminator (proBLUEVIEW, Cleaver scientific, Taiwan, China). Primers, PCR conditions, and electrophoresis conditions used in the study are listed in Table 1.

### 2.4. Statistical Analysis

All the experiments were repeated thrice. The chi-square and student’s *t*-test were used to test for statistical significance.

## 3. Results

### 3.1. Settings and Clinical Isolates

The tertiary care center for the study is a 500-bed teaching hospital. The BACTEC blood culture positivity rate was 15%. *S. epidermidis* was identified as Gram-positive, small, white, or greyish colonies, and catalase-positive, while DNAse was identified as negative, growing on blood agar and DNAse agar, respectively. *Rdr* gene’s species-specific target region further identified the isolates through PCR analysis [23,24] (Table 1). Of 150 CONS isolates, 75 (50%) were identified as *S. epidermidis*.

### 3.2. Screening and Presence of Complete, Missing, and Defective icaADBC Operon

All isolates of *S. epidermidis* were screened for the presence of *icaADBC* operon by detecting the *icaA* and *icaD,* and the operon was categorized as (1) Complete: both *icaA* and *icaD* detected, (2) Defective: only *icaA* or *icaD* detected, and (3) Missing: both *icaA* and *icaD* missing (Table 1). Out of 75 biofilm-positive isolates, the *icaADBC* operon was detected only in its complete form in 26 (34.6%) isolates, indicating that ~2/3 of clinically significant strains lacked this operon. Interestingly, only the *icaD* gene was detected in the defective operon while missing the *icaA*. The *icaA* was not detected alone and was always associated with *icaD* in the case of *icaA*-positive strains.

### 3.3. Activity of Complete, Missing, and Defective icaADBC Operon 

**Figure 1:**
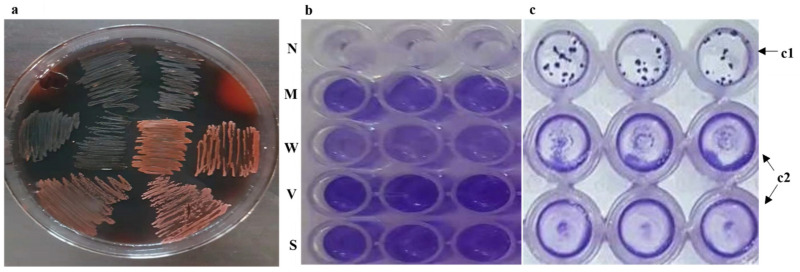
***icaADBC* operon activity, biofilm categories and biofilm morphotypes.** (**a**) Congo red assay; red: missing and defective *icaADBC* operon, Dry black: Actively complete *icaADBC* operon. (**b**) Microtiter-plate well assay; **N**: Negative control, **M**: Moderate, **W**: Weak, **V**: Very Strong, **S**: Strong. OD Cut off values: Biofilm negative: OD ≤ ODC, Weak positive: ODC < OD ≤ 2(ODC), Moderate positive: 2(ODC) < OD ≤ 4(ODC), Strong positive: 4(ODC) < OD ≤ 5(ODC) to 4(ODC) < OD ≤ 9(ODC), Very Strong positive: 9(ODC) < OD. (**c**) Biofilm morphotypes; **c1**: Aggregated microcolonies like biofilm, **c2**: Uniform confluent biofilm mass.

The *icaADBC* operon activity was not detected in the Congo red assay of the missing and defective operon categories. None of the isolates produced slime, and they were all Congo red negative (0% *icaADBC* activity), indicating that the operon could not synthesize the PNAG/PIA to detectable levels. In the case of the complete operon group (*n* = 26, 34.6%), the complete activity of the *icaADBC* operon and active synthesis of PIA was detected in 21 (80.7%) isolates (dry black colonies). It indicated that Congo red assay is a sensitive tool (80.7% sensitivity) for analyzing the *icaADBC* operon (PIA-dependent) biofilm-forming potential of *S. epidermidis*. Congo red positive activity was missing in 5 (19.3%) isolates (red/Bordeaux color colonies), although these isolates were encoding both *icaA* and *icaD* (Figure 1A). Thus, the complete operon was further categorized as actively complete (complete operon with positive Congo red assay). Out of 65 nosocomial *S. epidermidis* isolates, the polysaccharide-dependent biofilm-forming potential was only exhibited by 21 isolates (28%; *p* = 0.149), based on active *icaADBC* operon and synthesis of PIA/PNAG. 

### 3.4. Distribution of Genes Encoding MSCRAMMs 

All isolates of *S. epidermidis* were screened for the distribution of genes encoding MSCRAMMs. Out of 75 isolates, the genes encoding fibrinogen binding protein (*fbe*), extracellular matrix binding protein (*embp*), and laminin-binding protein (*eno*) were detected in 65 (86.6%), 63 (84%), and 69 (92%) isolates, respectively, and were categorized as group A. However, in group B, genes encoding the accumulation-associated protein (*aap*), biofilm homologous protein (*bhp*), and biofilm-associated protein (*bap*) were detected in 35 (46.6%), 18 (24%), and 1 (1.3%) isolate, respectively. The distribution of genes encoding MSCRAMMs among *S. epidermidis* is listed in Table 2 and in supplementary Figure S1.

### 3.5. Biofilm Formation Associated with the icaADBC Operon and MSCRAMMs

The 75 *S. epidermidis* isolates were checked for biofilm production in the humanized media under stress. *S. epidermidis* frequently produced biofilm (*n* = 65; 86.6%), and strong biofilm (Figure 1B) production was detected in 55 (84.6%) isolates. The contribution of the *icaADBC* operon and the role of PIA/PNAG in biofilm formation were not significant (*n* = 21; 28%; *p* = 0.149) (Table 3). However, the association of MSCRAMMs with biofilm formation (Table 2) showed that MSCRAMMs might play an active and significant role in the PIA-independent mechanism (*n* = 59; 90.7%; *p* = 0.007) adopted by *S. epidermidis*. It is worth noting that all isolates encoding complete and the actively complete *icaADBC* operon were also encoding both *fbe* and *embp* (*n* = 26; 100%), except for one isolate lacking the *embp*. 

### 3.6. Screening for IS256 and Association with Biofilm Formation and icaADBC Operon

Of the 65 biofilm-positive *S. epidermidis*, 25 isolates encoded the *IS256*, 24 (43.6%) of which belonged to the strong biofilm producers’ group (including 17 very strong biofilm-producing isolates), 1 belonged to moderate while 1 isolate, carrying *IS256,* belonged to biofilm negative groups. None of the weak biofilm-producing isolates were encoding this gene, while it was detected in three isolates classified as slow growers. However, the association of *IS256* with biofilm formation was statistically non-significant (*n* = 25; 38.4%; *p* = 0.925).

Of the 21 actively complete *icaADBC* operon encoding isolates, 10 (47.6%) isolates were associated with *IS256*, while 11 isolates lacked this insertion sequence. Similarly, of the 5 complete *icaADBC* operon groups, which could not synthesize detectable PIA, the *IS256* was present in 4 (80%; *p* = 0.191) isolates. With the MSCRAMMs positive group, *IS256* was randomly distributed among these isolates.

### 3.7. Association of icaADBC Operon and MSCRAMMs with Biofilm Mass Density

To check biofilm determinants’ effect on biofilm mass and density, cutoff OD values ≥ 1.7 and ≤2.2 (highest OD obtained in this study) were selected, and the isolates were categorized into the following groups: (1-) isolates encoding the MSCRAMMs *fbe* and *embp*, and actively complete *icaADBC* operon (*n* = 21; 32%). (2-) isolates encoding only the MSCRAMMs *fbe*+ *embp* (*n* = 20; 31%). In group 1, four isolates of the strong biofilm producers obtained OD ranging from 1.7 to 2.1, while the density of the rest of the isolates was <1.7, randomly distributed between 0.8 and 1.6. In the second group, three isolates obtained this cut-off range, while the OD of the rest of the isolates was <1.7. 

### 3.8. Appearance of Biofilm Morphotypes (BM) and Association with icaADBC Operon and MSCRAMMs

Two unique types of BM (Figure 1C) appeared in the base wells of 96 well microtiter plates: 1 – uniform confluent biofilm mass, regularly distributed in the base, and 2 – aggregated microcolonies like biofilm formation, randomly distributed in the microtiter well base. The association of the biofilm determinants with biofilm morphotypes was checked. All biofilm-positive isolates, encoding only the MSCRAMMs, while lacking the actively complete *icaADBC* operon, were uniquely distributed in the first group only, and none of these isolates formed microcolonies like biofilm morphology. Isolates encoding both actively complete *icaADBC* operon and MSCRAMMs were randomly distributed in both groups, with 50% of the isolates encoding the actively complete *icaADBC* operon producing the microcolonies morphotypes, demonstrating the role of the PIA in strong adhesive aggregated microcolonies-like appearance of biofilm formation.

## 4. Discussion

*S. epidermidis* is an opportunistic and frequently isolating pathogen in pediatric bacteremia. Nosocomial *S. epidermidis* has been the main isolate from bacteremia samples throughout previous studies from children and neonates [25,26,27]. In the current study, the high prevalence of blood-borne *S. epidermidis* highlighted its clinical significance. It indicates that this opportunistic pathogen is the leading cause of bacteremia in pediatrics and can significantly increase hospital stay and morbidity with subsequent consequences. In *S. epidermidis*, the polysaccharide-dependent biofilm formation is significantly replaced by a polysaccharide-independent mechanism. It might be because the PIA synthesis is an energy-consuming process [28], thus it is less suitable for infection in invasive conditions in high-shear environments e.g., bacteremia. The MSCRAMMs are abundantly present in isolates producing strong biofilms, while lacking the polysaccharide-synthesizing machinery for biofilm formation. A shift to polysaccharide-independent mechanism might explain the pathogenesis of nosocomial *S. epidermidis*. 

The *icaADBC* operon is responsible for PIA-dependent biofilm formation in *S. epidermidis*. Based on the detection of *icaA* and *icaD*, the complete *icaADBC* operon was missing in 2/3 of strains, and its actual contribution (actively complete operon) further diminished to <1/3 (*p* = 0.149). These findings show that the simultaneous presence of *icaA* and *icaD* are markers of the complete expression and actively synthesizing PIA strains since *icaA*-positive cases were consistently associated with *icaD*. Hence, only the detection of *icaA* represented the actively complete operon with 80.7% sensitivity.

The findings of this study contradict previous findings regarding the association of *icaADBC* operon with Congo red activity. The PIA-dependent biofilm-forming potential and higher levels of Congo red activity has been reported in *S. epidermidis,* even in the absence of the *icaADBC* operon [25,29,30]. Positive Congo red activity in the absence of PIA, which is the only Congo red detectable exopolysaccharide encoded by *icaADBC* operon [31], makes these findings unreliable. Our results align with the findings of two previous studies, which reported 23% and 25% Congo red activity [32,33]. Other studies also suggested that Congo red agar cannot be recommended alone for the detection of Staphylococcal biofilms [34,35]. The current findings show that Congo red assay is a sensitive technique (80.7% sensitivity) for detecting only the polysaccharide-dependent biofilm formation potential of *S. epidermidis*. 

In this current study, the prevalence and overall contribution of the *icaADBC* operon in biofilm formation were low (*n* = 21; 32.3%; *p* = 0.149), indicating PIA-independent solid alternative strategies adopted by *S. epidermidis*. The findings also show that group A MSCRAMMs were significantly (*fbe; p* = 0.007, *embp; p* = 0.026) associated with biofilm-producing *S. epidermidis*, of which 55 isolates (84.6%) were biofilm strong positive; group B (*aap; p = 0*.069, *bhp; p* = 0.633, *bap*; *p* = > 0.05) and a member of group A (*eno*; *p* = 0.802) were statistically non-significant. The findings of this study are in line with findings of previous studies that reported a high prevalence of group A MSCRAMMs (*fbe* and *embp)* in clinical *S. epidermidis* [29,30]. However, unlike the previous findings [36,37,38,39], the prevalence of MSCRAMMs classified in group B in the current study is low and has a non-significant association with biofilm-positive isolates. The findings of this current study suggest that MSCRAMMs are not distributed equally among isolates from pediatric wards and NICU, and this indicates high clonal variations among nosocomial *S. epidermidis* strains in this group of patients. 

Interestingly, research has shown that *fbe,* one of the most prevalent MSCRAMMs in this study, is expressed only during in-vivo conditions, induced by host-specific signals [40]. To mimic the in-vivo conditions and as stress inducers, 5% human blood plasma and 5% glucose were added to the biofilm assay and obtained as high as 86.6% biofilm positive *S. epidermidis* isolates, of which 84.6% were strong biofilm positive. More interestingly, these isolates were all encoding *fbe* and *embp*. Our findings are supported by previous studies, where the addition of plasma and blood components significantly induced MSCRAMMs gene expression and increased Staphylococcal biofilm formation [41,42,43,44]. The current study suggests that this class of MSCRAMMs was induced by the addition of human blood plasma and glucose, and potentially contributed to its role in biofilm formation. However, further molecular studies are recommended to validate these results. The fibrinogen binding protein and extracellular matrix-binding proteins might be potential anti-biofilm drug targets, markers of rapid diagnosis, and potential vaccine candidates of *S. epidermidis* involved in pediatric bacteremia.

*IS256* is an insertion sequence element, previously known to affect biofilm formation and disruption of the Staphylococcal *icaADBC* operon by insertion/excision mechanism [45] and co-existed with clinically significant biofilm-positive *S. epidermidis,* making it a pathogenicity marker [46]. An unexpected association of *IS256* with biofilm formation and slime synthesis was obtained in this study. The prevalence of *IS256* (*n* = 25; 38.4%; *p* = 0.925), and association with actively complete *icaADBC* operon (slime production) (*n* = 10; 47.6%; *p* = 0.294) were statistically non-significant in the clinical isolates under study, thus *IS256* is not a significant regulator of the *icaADBC* operon. However, its association with strong biofilm formation (*n* = 24; 43.6%; *p* = 0.044) was so statistically significant and unexplainable. We, therefore, suggest further higher-level molecular studies regarding the positive regulatory effect of *IS256* on strong biofilm formation. Similarly, no statistical significance (*n* = 4; 80%; *p* = 0.191) was found with complete *icaADBC* operon (*icaA* and *icaD* +ve; Congo red assay −ve) encoding isolates. This correlation is in line with the findings of Arciola and colleagues, who stated that switching on/off *ica* locus is not naturally dependent on the insertion/excision of *IS256* [47]. 

Findings of the current study showed no statistically significant difference (*p* > 0.05) in biofilm density among strains of *S. epidermidis* encoding either actively complete *icaADBC* operon alone or in combination with MSCRAMMs. Indeed, biofilm regulation phenomena, such as *agr* quorum sensing mechanisms and *psms* (phenol soluble modulines), have been detected and analyzed extensively in *S. epidermidis* [48,49] and are proposed to positively regulate the overgrowth of both polysaccharide and MSCRAMMs-dependent biofilm formation under the induction conditions followed in this study. In this current study, the *icaADBC* operon encoding isolates produced aggregated microcolonies like biofilm morphotypes in humanized medium supplemented with a 5% glucose stress inducer. Previous findings support our results. Dobinsky and coworkers [50] observed macroscopic cellular aggregation from the production and induction of PIA by glucose. Cramton and coleagues [51] also reported that the micro and macro-colony formation stage in biofilm depends on bacterial inter-cellular aggregation, primarily mediated by PIA. The current study has few limitations. It is a single-center experience, limited only to pediatrics department including pediatrics wards and nursery ICU. Extensive study of multiple tertiary care centers is recommended. Studies on MSCRAMMs mutant strains are recommended to verify their contribution in polysaccharide-independent biofilm formation.

## 5. Conclusions

*IcaA* is the representative module of an actively complete *icaADBC* operon. The *icaADBC* operon component is not vital for biofilm formation. Nosocomial *S. epidermidis* has significantly adopted PIA-independent biofilm-forming strategies, and highly prevalent ‘group A’ MSCRAMMs might be potentially involved in such mechanisms. The microtiter-plate well assay is a recommended tool for detecting biofilm formation in strains using either PIA-dependent or PIA-independent mechanisms. 

## Figures and Tables

**Table 1:** Primers for virulence determinants.

Name	Abbreviation	Primers (5ʹ 3ʹ)	Ampli-Con Size (bp)
*S*. *epidermidis species specific*	*Rdr*	F: AAGAGCGTGGAGAAAAGTATCAAG R: TCGATACCATCAAAAAGTTGG	130
* Intercellular adhesin D	*icaD*	F: ATGGTCAAGCCCAGACAGAG R: CGTGTTTTCAACATTTAATGCAA	198
Insertion Sequence 256	*IS256*	F: AGTCCTTTTACGGTACAATG R: TGTGCGCATCAGAAATAACG	762
** Intercellular adhesin A	*icaA*	F: TCTCTTGCAGGAGCAATCAA R: TCAGGCACTAACATCCAGCA	188
Laminin binding Protein/Enolase	*Eno*	F: ACGTGCAGCAGCTGACT R: CAACAGCATCTTCAGTACCTTC	301
Fibrinogen binding Protein	*fbe/SdrG*	F: CTACAAGTTCAGGTCAAGGACAAGG R: GCGTCGGCGTATATCCTTCAG	273
Accumulation associated protein	*Aap*	F: AAACGGTGGTATCTTACGTGAA R: CAATGTTGCACCATCTAAATCAGCT	465
Bap homologue Protein	*Bhp*	F: ATGGTATTAGCAAGCTCTCAGCTGG R: AGGGTTTCCATCTGGATCCG	1585
Extracellular matrix binding protein	*Embp*	F: AGCGGTACAAATGTCAAT R: AGAAGTGCTCTAGCATCATCC	455
Biofilm associated protein	*Bap*	F: CCCTATATCGAAGGTGTAGAATTG R: GCTGTTGAAGTTAATACTGTACCTGC	971

* *mecA* ** *TSST-1* (intended for publication in preparation) included in these multiplex groups.

**Table 2:** Distribution of MSCRAMMs/CWA among biofilm positive and negative *S. epidermidis*.

MSCRAMMs/CWA	Biofilm Positive					Biofilm Negative		*p*-Values
	In Biofilm Positive Isolates *n* (%)	Total Strong *n* (%) 55 (84.6%)		Moderate *n* (%)	Weak *n* (%)	Negative *n* (%)	SG * n (%)	
		V. Strong *n* (%)	Strong *n* (%)					
*fbe* ^a^	59 (90.7%)	11 (84.6%)	42 (100%)	5 (71.4%)	1 (33.3%)	4 (80%)	2 (40%)	0.007
*embp* ^a^	57 (87. 6%)	10 (77%)	42 (100%)	4 (57.1%)	1 (33.3%)	4 (80%)	2 (40%)	0.026
*eno* ^b^	60 (92.3%)	11 (84.6%)	42 (100%)	5 (71.4%)	2 (66.6%)	5 (100%)	4 (80%)	0.802
*aap* ^c^	33 (50.7%)	5 (38.4%)	25 (59.5%)	2 (28.5%)	1 (33.3%)	2 (40%)	0 (0%)	0.069
*bhp* ^c^	15 (23%)	4 (30.7%)	11 (26.1%)	0 (0%)	0 (0%)	2 (40%)	1 (20%)	0.633
*bap* ^c^	1 (1.5%)	0 (0%)	1 (2.3%)	0 (0%)	0 (0%)	0 (0%)	0 (0%)	NA
**Total isolates (*n* = 75)**	65 (86.6%)	13 (20%)	42 (64.6%)	7 (10.7%)	3 (4.6%)	5 (7.6%)	5 (7.6%)	NA

* SG: Slow growers. *p* < 0.05 was considered significant. **^a^**: *fbe* and *embp* are the only MSCRAMMs statistically significant in biofilm formation and especially are associated with biofilm strong positive. **^b^**: *Eno* is present in 92% biofilm positive isolates; however, *p*-value is >0.05 so statistically non-significant. **^c^**: MSCRAMMs statistically non-significant in biofilm. NA: Not applicable.

**Table 3:** Distribution and Activity of *icaADBC* operon among biofilm positive and negative *S. epidermidis*.

*icaADBC* *Operon*	Biofilm Positive (*n* = 65)					Biofilm Negative (*n* = 10)		*p*-Values
	In Biofilm Positive Isolates *n* (%)	Total Strong *n* (%) 55 (84.6%)		Moderate *n* (%)	Weak *n* (%)	Negative *n* (%)	SG * n (%)	
		V. Strong *n* (%)	Strong *n* (%)					
**Complete ^a^** **(Actively complete) ^b^**	26 (40%) (21 (80.7% ** & 28% ***))	5 (19%) 4 (19%)	19 (73%) 15 (71.4%)	2 (7.6%) 2 (9.5%)	0 (0%) 0 (0%)	0 (0%) 0 (0%)	1 (3.8%) 1 (4.7%)	.065 0.149
**Defective ^c^**	15 (23%)	2 (13.3%)	11 (73.3%)	0 (0%)	2 (13.3%)	2 (13.3%)	2 (13.3%)	0.252
**Missing ^d^**	24 (37%)	0 (0%)	16 (66.6%)	6 (25%)	2 (8.3%)	3 (8.8%)	2 (5.8%)	0.429
**Total isolates** **(*n* = 75)**	65 (86.6%)	13 (20%)	42 (64.6%)	7 (10.7%)	3(4.6%)	5 (7.6%)	5 (7.6%)	NA

**^a^** *icaA* & *icaD* + ve, **^b^** *IcaA* & *icaD* + ve CRA + ve, **^c^** *icaA* − ve *icaD* + ve CRA − ve, **^d^** *icaA* & *icaD* − ve CRA − ve, * Slow Growers, ** % of 26, *** % of 65. *p* < 0.05 was considered significant. Actively complete operon indicates positive activity in Congo red assay. The *p*-values indicate that all categories of *icaADBC* operon are statistically not significant in biofilm. NA: Not applicable.

## Data Availability

Data will be made available on request.

## Supplementary Materials

The following supporting information can be downloaded at: https://www.mdpi.com/article/10.3390/medicina58111510/s1, Figure S1: Representative virulence genes profiling of *S. epidermidis*.
