# Supplementary material for: Staphylococcus epidermidis Pathogenesis: Interplay of icaADBC Operon and MSCRAMMs in Biofilm Formation of Isolates from Pediatric Bacteremia in Peshawar, Pakistan
Source: Medicina (Kaunas). 2022 Oct 24;58(11):1510. doi: 10.3390/medicina58111510 (PMC9696285; doi:10.3390/medicina58111510)
Supplement: Supplementary file 1 [file medicina-58-01510-s001.zip › medicina-1951692-supplementary.pdf]

medicina

MDPI

Supplementary Materials

Staphylococcus epidermidis Pathogenesis: Interplay of *icaADBC* Operon and MSCRAMMs in Biofilm Formation of Isolates from Pediatric Bacteremia in Peshawar, Pakistan

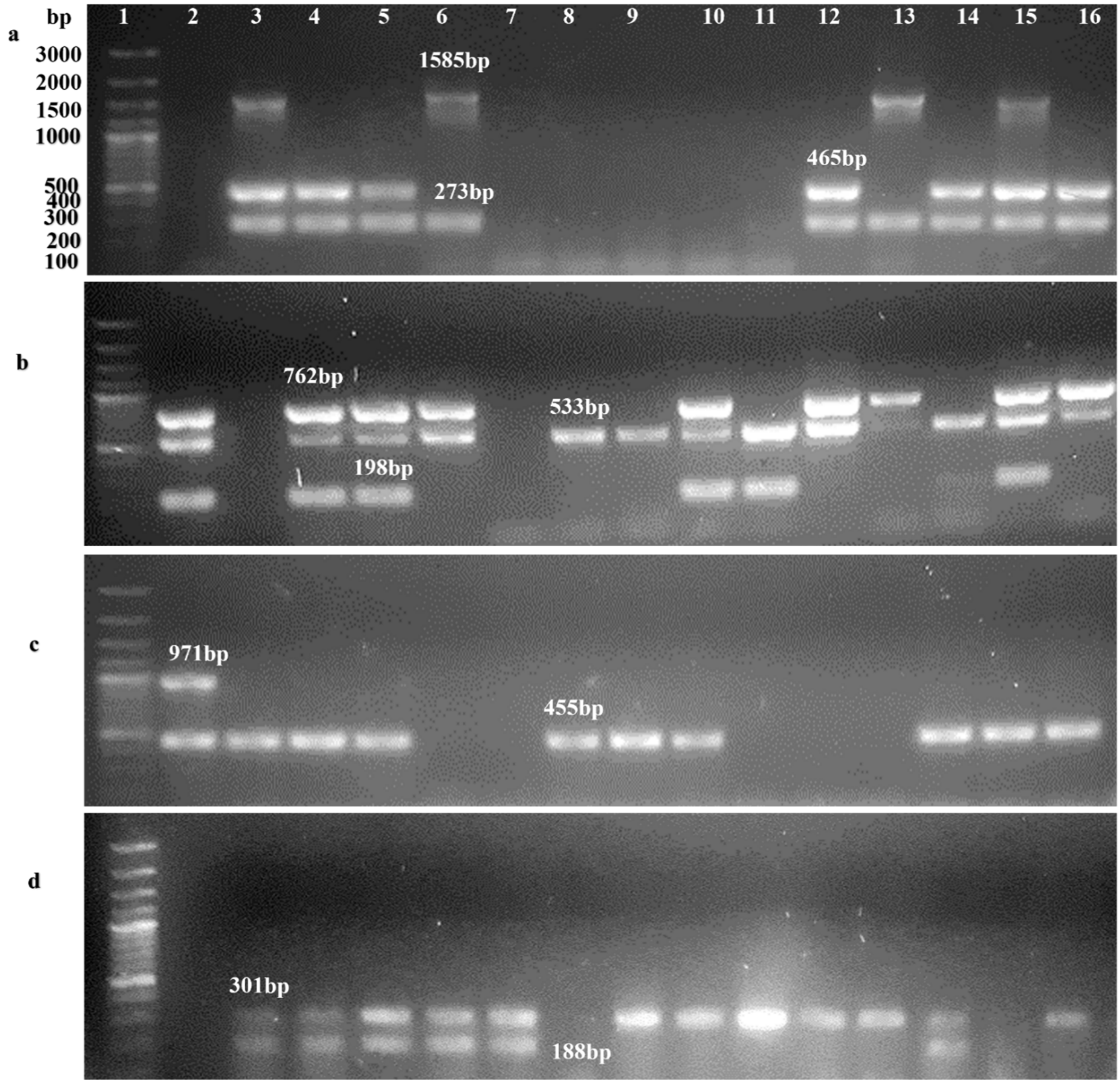

Figure S1. Representative virulence genes profiling of *S. epidermidis*. (**a**) 2: negative control, 3: positive control, *bhp* (1585bp), *aap* (465bp), *fbe* (273bp); (**b**) 2: positive control, 3: negative control, *IS256* (762bp), *mecA* (533bp, not part of the manuscript/intended for another publication), *icaD* (198bp); (**c**) 2: positive control, *bap* (971bp), *embp* (455bp). (**d**) *eno* (301bp), *icaA* (188bp); Multiplex PCR amplification of representative isolates. 1.5% agarose gel; 1 (100bp DNA Marker); 4-16: test isolates; Time: 110 minutes; Voltage: 75; Buffer: TAE 1X.
